# Model of Care for Microelimination of Hepatitis C Virus Infection among People Who Inject Drugs

**DOI:** 10.3390/jcm10174001

**Published:** 2021-09-03

**Authors:** Francesco Giuseppe Foschi, Alberto Borghi, Alberto Grassi, Arianna Lanzi, Elvira Speranza, Teo Vignoli, Lucia Napoli, Deanna Olivoni, Michele Sanza, Edoardo Polidori, Giovanni Greco, Paolo Bassi, Francesco Cristini, Giorgio Ballardini, Mattia Altini, Fabio Conti, on behalf of MITH Group

**Affiliations:** 1 Internal Medicine Department, Faenza Hospital, 48018 Faenza, Italy; francesco.foschi@auslromagna.it (F.G.F.); alberto.borghi@auslromagna.it (A.B.); lucia.napoli@auslromagna.it (L.N.); 2 Internal Medicine Department, Rimini Hospital, 47923 Rimini, Italy; alberto.grassi@auslromagna.it (A.G.); giorgio.ballardini@auslromagna.it (G.B.); 3 Mental Health and Pathological Addictions Department, Addiction Treatment Service of Cesena, 47521 Cesena, Italy; arianna.lanzi@auslromagna.it (A.L.); michele.sanza@auslromagna.it (M.S.); 4 Mental Health and Pathological Addictions Department, Addiction Treatment Service of Faenza, 48018 Faenza, Italy; elvira.speranza@auslromagna.it; 5 Mental Health and Pathological Addictions Department, Addiction Treatment Service of Lugo, 48121 Ravenna, Italy; teo.vignoli@auslromagna.it (T.V.); deanna.olivoni@auslromagna.it (D.O.); giovanni.greco@auslromagna.it (G.G.); 6 Mental Health and Pathological Addictions Department, Addiction Treatment Service of Rimini and Forlì, 47121 Forlì, Italy; edoardo.polidori@auslromagna.it; 7 Infectious Disease Department, Ravenna Hospital, 48121 Ravenna, Italy; paolo.bassi@auslromagna.it; 8 Infectious Disease Department, Rimini Hospital, 47923 Rimini, Italy; francesco.cristini@auslromagna.it; 9 Local Healthcare Authority of Romagna, AUSL Romagna, 48121 Ravenna, Italy; mattia.altini@auslromagna.it

**Keywords:** HCV treatment, HCV microelimination, drug users, reinfection, people who inject drugs, direct acting antivirals

## Abstract

Background: People who inject drugs (PWID) are the largest group at risk for HCV infection. Despite the direct acting antivirals (DAA) advancements, HCV elimination has been hindered by real-life difficulties in PWID. Aims: This study aimed to assess the impact of a multidisciplinary intervention strategy where HCV screening, treatment and follow-up were performed at the same location on efficacy and safety of DAA-therapy in real-life PWID population. Methods: All HCV-infected PWID referred to five specialized outpatient centers for drug addicts (SerDs) in Northern Italy were prospectively enrolled from May 2015 to December 2019. Hepatologists and SerDs healthcare workers collaborated together in the management of PWID inside the SerDs. Sustained virologic response (SVR), safety of treatment, proportion of patients lost to follow-up and reinfection rate were evaluated. Results: A total of 358 PWID started antiviral treatment. About 50% of patients had advanced fibrosis/cirrhosis, 69% received opioid substitution treatment, and 20.7% self-reported recent injecting use. SVR was achieved in 338 (94.4%) patients. Two patients died during treatment; one prematurely discontinued, resulting in a non-responder; twelve were lost during treatment/follow-up; and five relapsed. No serious adverse events were reported. SVR was lower in recent PWID than in former ones (89.2% vs. 95.8%; *p* = 0.028). Seven reinfections were detected, equating to an incidence of 1.25/100 person-years. Reinfection was associated with recent drug use (OR 11.07, 95%CI 2.10–58.38; *p* = 0.005). Conclusion: Our embedded treatment model could be appropriate to increase the linkage to care of HCV-infected PWID. In this setting, DAA regimens are well tolerated and highly effective, achieving a lower rate of reinfection.

## 1. Introduction

It is well established that hepatitis C virus (HCV) is a major cause of liver related mortality and a well-recognized trigger for extrahepatic disease [1]. In 2015, it was estimated that viremic HCV affected 71.1 million people, i.e., 1% of the global population [2]. The landscape has dramatically changed after the introduction of all-oral direct-acting antivirals (DAAs) in 2013. Manageable, safe, and effective widespread use of DAAs has permitted the treatment in a large part of the HCV population, including both individuals with and without cirrhosis [3].

In 2016, the World Health Organization (WHO) recognized HCV as a major health problem, and since then, it has aimed to developed better strategies to eradicate HCV by 2030 [4]. With the availability of high-efficacy DAAs, these strategies are no longer intended as an improvement of therapeutic effectiveness but an expansion of public policies of screening and treatment (increasing HCV diagnoses from <20% to 90% and the number of eligible persons receiving treatment from <10% to 80%), contemporarily reducing new infections and HCV deaths [4].

People who inject drugs (PWID) are a key population for microelimination strategies; however, data are limited on best practices for engagement in HCV care. Today, more than 4 million European people are PWID, and 53.2–64.7% of them are HCV carriers [5]; moreover, 23% of new HCV infections occur among recent PWID. The high risk of environmental exposure of PWID explains the higher prevalence of HCV rather than in the general population; incarceration, sex workers, unstable housing, and homelessness are experiences connected to dangerous sex practice and use of unsafe needle for drug injection, the leading cause of HCV transmission [5].

In the interferon-based regimens era, PWID were rarely treated for HCV, because the adherence to therapy was poor, and sustained virologic response (SVR) was difficult to attain. Today, DAAs are as safe and effective in in general population as in PWID, even in ongoing drug addiction or opioid substitution treatment (OST) [6,7,8]. Despite this evidence, inequalities continue in access to HCV treatment, both for the scarce awareness of the problem among PWID and for heterogeneous screening policies in addiction clinics [9]. Likewise, there is an unbalance between diagnosis of HCV in drug-addiction services and referral to hepatologic centers, due to social, clinical, and health system barriers [10,11]. In some countries, policies of facilitation inside addiction clinics have shown benefits, increasing the screening for HCV and addressing PWID to antiviral therapy [12]. In Italy, PWID are usually managed in specialized outpatient centers for drug addicts (SerDs) by toxicologists, psychiatrists, and nurses skilled in the management of patients with substance-use disorders who take care of all the clinical aspects related to addiction. As a consequence, an interdisciplinary collaborative program was initiated between neighboring SerDs of North Italy and territorial centers for liver diseases in order to improve screening and therapeutic policies according to WHO indications.

The aim of this study was to evaluate if our management policy could improve HCV treatment uptake and outcomes in a cohort of PWID and to propose a simplified linkage-to-care model to effectively treat HCV infection. In this regard, we analyzed SVR, treatment completion, adherence, safety, and HCV reinfection of interferon-free therapies.

## 2. Materials and Methods

### 2.1. Study Population

From May 2015 to December 2019, PWID with chronic hepatitis C (CHC), referring to five SerDs in Emilia-Romagna, Italy (Faenza, Lugo, Rimini, Cesena, Ravenna), were prospectively enrolled. Recruitment started at different time points at different sites. PWID were defined as patients with a history of injecting drug use (reported injecting heroin or cocaine use ever). Patients who self-reported injection drug use within 6 months prior to screening were considered recent PWID, while all others were referred to as former PWID. Patients with decompensated cirrhosis or diagnosis of hepatocellular carcinoma (HCC); patients with heart, kidney, and pulmonary failure; pregnant women; and people aged <18 years were excluded. Subjects with HIV co-infection were referred to another infectious disease service and were excluded from this study. Treatment with sofosbuvir alone and sofosbuvir/simeprevir were defined as first-generation regimens; those with sofosbuvir/daclatasvir, sofosbuvir/ledipasvir, or ombitasvir/paritaprevir/ritonavir and dasabuvir as second-generation regimens; and those with glecaprevir/pibrentasvir, sofosbuvir/velpatasvir, or grazoprevir/elbasvir as third-generation regimens.

The study protocol was approved by our local Ethic Committee (Comitato Etico della Romagna, C.E.Rom., approval code: 7723/2019 I.5/206, registration number 2496) and was conducted in compliance with the Declaration of Helsinki and with the local Review Board regulations.

### 2.2. Intervention Model and Study Assessment

**Figure 1:**
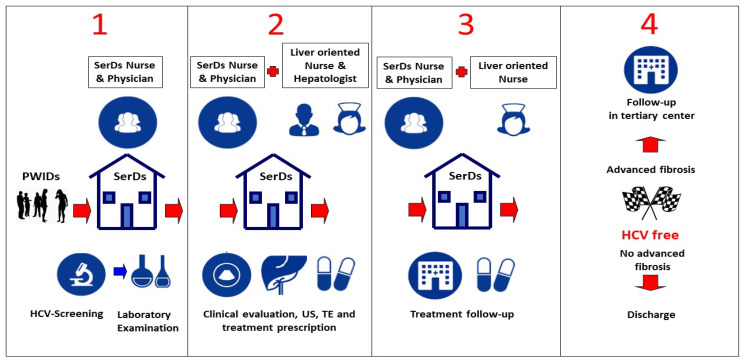
Model of care representing multidisciplinary approach in SerDs. SerDs, specialized outpatient centers for drug addicts; PWID, people who inject drugs; US, ultrasonography; TE, transient elastography.

All SerDs shared the same clinical approach as depicted in Figure 1.

Step 1: When an HCV-infected subject was identified by SerDs healthcare workers, they underwent complete blood tests, including viral load, genotyping, HIV, and HBV.

Step 2: All patients with CHC were referred to comprehensive multidisciplinary care, including nursing, medical care, and logistic support performed by liver-oriented nurses and hepatologists or infectious-disease physicians. The first visit with an HCV specialist was performed within two months from detection of HCV-RNA positivity in the SerDs. Within each SerD, patients habitually received nursing/medical care and pharmacological/psychological support. On the same day, all patients had access to clinical evaluation, diagnostic procedures (liver ultrasonography and transient elastography), and prescription of HCV treatment. The choice of DAA and treatment duration for eligible patients was performed according to EASL guidelines and our Italian policies established by the Italian Drug Regulatory Agency available at the time of enrolment. During the first part of the study period, access to all oral DAA therapy for HCV in Italy was restricted on the basis of fibrosis severity or the presence of life-threatening extra hepatic disease. Before initiating treatment, patients were educated on HCV and their chosen regimen by liver-oriented nurses.

Step 3: All subjects were followed-up on a monthly basis during treatment in the SerDs. The occurrence of serious adverse events (SAEs) leading to temporary or permanent treatment discontinuation, hospitalization, or death was prospectively recorded by the referring physician. Sustained virologic response (SVR) was defined as plasma HCV-RNA below the limit of detection 12 weeks after the end-of-treatment (EOT). Relapse was defined as a confirmed HCV-RNA ≥ 12 IU/mL value during follow-up in patients with undetectable HCV-RNA at the EOT. Reinfection was defined as a single positive HCV-RNA test measured after SVR and a difference in HCV genotype or subtype or a significantly different clustering located in different clades in the phylogenetic tree.

Step 4: Post-SVR, patients were followed up through standard of care. Patients with advanced fibrosis and/or who develop risk factors from a different cause (such as non-alcoholic steatohepatitis or alcoholic liver disease) underwent regular check-up every 6 months in a tertiary care center to monitor HCV reinfection and other comorbidities, such as cirrhosis decompensation and incidence of HCC. Other patients returned to SerDs physician’s surveillance twelve months after SVR, monitoring for HCV reinfection through six-monthly HCV-RNA assessment.

### 2.3. Clinical and Virological Assessment

Assessments at enrolment included information on demographic characteristics (age, sex, and BMI), drug and alcohol use, injecting risk behaviours, psychiatric history, comorbidities, and drug treatment. All patients enrolled were submitted to clinical evaluation and routine laboratory assays for liver functionality. Ultrasound was performed at baseline to exclude the presence of HCC. Stage of liver fibrosis was assessed by transient elastography by Fibroscan. The chosen cutoff values for significant fibrosis, advanced fibrosis, and cirrhosis were 7.1, 9.5, and 12.5 kPa, respectively [13]. In participants who lacked liver stiffness measurements, the stage of liver disease was assessed by using the Fibrosis-4 (FIB-4) index.

### 2.4. Statistical Analysis

As primary outcome, the effectiveness of the collaboration of the SerDs and liver-oriented teams was tested by calculating (a) the rate of PWID who started the HCV therapy, (b) the proportion of patients who achieved SVR, and (c) the reinfection rate. SVR was evaluated according to intention-to-treat (ITT) analysis, which included all patients who received at least one dose of the study drug, considering all missing data at the date of SVR assessment as failures. Subgroup analyses were conducted in the modified ITT (mITT) population, which excluded patients with non-virologic failure (SVR non-response due to early discontinuation, death, and/or lost to follow-up). The incidence of reinfection was calculated as the number of occurrences per 100 person years (PY) of follow-up. The secondary outcome was treatment adherence, which was defined as taking at least one pill but not completing the 80% of the planned course. Continuous variables were expressed as median (range) and categorical variables as number (%). The Chi-square test or the Fisher’s exact test, when appropriate, was used to compare proportions among treatment groups. Multivariate analysis was performed by using logistic regression to evaluate independent factors associated with failure to achieve SVR and reinfection after DAA. Data were analyzed by using IBM SPSS 24.0 version (IBM Corporation, Somers, NY, USA). Statistical significance was defined when *p* < 0.05 in a two-tailed test with a 95% confidence interval.

## 3. Results

### 3.1. Patient Characteristics

**Figure 2:**
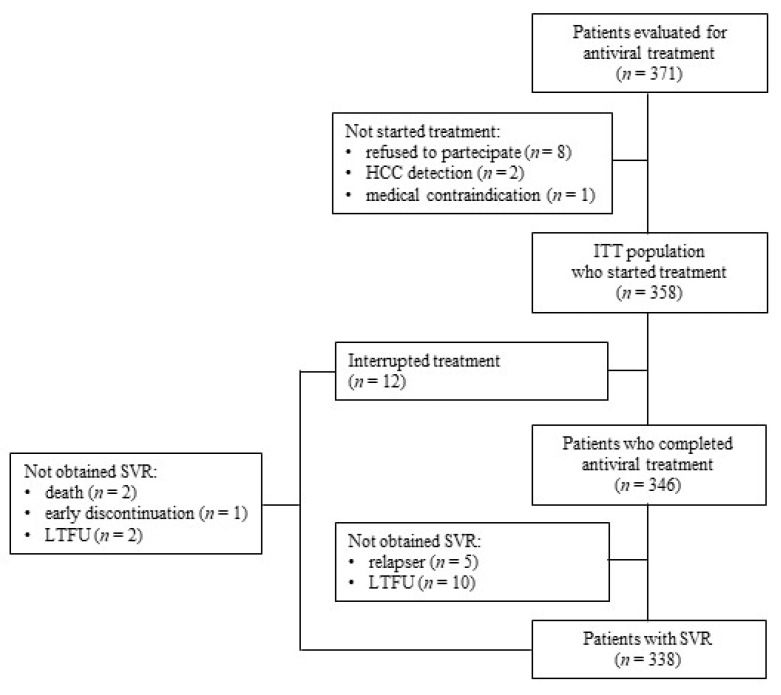
Derivation of the analysis population. ITT, intention-to-treat; LTFU, loss to follow-up; SVR, sustained virologic response; mITT, modified intention-to-treat.

A total of 371 HCV-infected PWID were evaluated for antiviral treatment, and 358 (96.5%) started interferon-free DAA combinations (Figure 2). Eight patients declined care and were excluded. One patient withdrew from treatment due to neurosurgery. During screening, ultrasound HCC was found in two patients, and antiviral therapy was postponed until after HCC eradication.

Baseline demographics and behavioral and clinical characteristics of the participants are reported in Table 1. The median age was 49 (18–69) years. A strong prevalence of male sex (83%) was observed, and advanced fibrosis or cirrhosis was present in 49.1% of patients. About 20% were treatment experienced, all with PEGylated interferon. Two hundred-forty-seven (69%) received the OST with methadone or buprenorphine (82.6% and 17.4%, respectively) and 74 (20.7%) self-reported recent injecting drug use. Alcohol consumption was observed in 259 (72.3%) patients, and 145 of them had a history of alcohol abuse. The most frequent HCV genotypes were 1a and 3 (41.1% and 39.7%, respectively). HBV co-infection was found only in four (1.1%) patients. One hundred and forty (39.1%) presented a psychiatric disorder, most frequently being sleep disorders, depression, and anxiety. Third-generation regimens were chosen for HCV therapy in 83.5% of patients, and the main DAA regimen used was glecaprevir/pibrentasvir (65%).

### 3.2. Treatment Completion, Adherence, and Efficacy

Overall, 346/358 (96.6%) participants completed treatment (Figure 2). Two patients died: one due to myocardial infarction after 16 weeks of treatment with sofosbuvir/daclatasvir that was unrelated to the antiviral medication, and one due to an unknown cause after 3 weeks of treatment with glecaprevir/pibrentasvir. In one patient, treatment with sofosbuvir/ribavirin was stopped after 9 weeks, due to decompensation of liver disease and resulted non responder. A further two patients were lost during treatment, and data on viral load were not available. Seven patients voluntarily discontinued the therapy but still showed SVR. Ten patients obtained EOT response but failed to return for follow-up evaluation (including two incarceration) missing SVR data.

No severe adverse events were reported, and treatment was generally well tolerated. Only mild adverse episodes were reported during treatment: fatigue being the most common (31%), followed by headache (25.1%), insomnia (19.8%), and nausea (7%).

According to ITT analysis, SVR was achieved in 338/358 patients (94.4%; 95%CI 91.5–96.6). Five patients relapsed (three with advanced fibrosis received first/second-generation regimens, one with cirrhosis received sofosbuvir/velpatasvir, and one with significant fibrosis received glecaprevir/pibrentasvir). The overall SVR rates by genotypes were as follows: 96% (95%CI 91.4–98.5) in GT1a, 90.5% (95%CI 69.6–98.8) in GT1b, 93% (95%CI 87.4–96.6) in GT3, and 95.5% (95%CI 84.5–99.4) in GT4 (Table 2). The recent PWIDs achieved an SVR rate of 89.2%% (95%CI 79.8–95.2) compared with 95.8% (95%CI 92.7–97.8) in the former PWIDs (*p* = 0.028) (Table 3). However, loss to follow-up was more frequent in the group of recent PWID (9.5% vs. 1.8%; *p* = 0.004). SVR rates were similar in patients receiving and not receiving OST: 93.5% (95%CI 89.7–96.2) and 96.4% (95%CI 91–99), respectively (*p* = 0.273). No significant differences were observed in SVR rates for any other patient characteristic subgroup. Subjects treated with a first or second-generation regimen had a higher likelihood of treatment failure than those treated with third-generation antivirals, but this difference did not reach statistical significance (*p* = 0.291). Using multivariate analysis, only recent drug use was independently associated with treatment failure (OR 2.747, 95%CI 1.080–6.992; *p* = 0.034) (Table 3). The characteristics of the patients according to their history of injecting drug use at the date of starting DAA therapy are summarized in Supplementary Materials Table S1.

The mITT population included 343 patients, and SVR was achieved in 338 (98.5%; 95%CI 96.6–99.5). No significant differences were observed in SVR rates between patients aged < or ≥50 years, males of females, and recent and former drug users, regardless of whether they were pretreated, cirrhotic, alcohol users, or had psychiatric comorbidities. The proportion of SVR was higher among patients receiving OST compared with non-OST (99.6% vs. 96.4%, *p* = 0.039) and patients treated with third-generation regimens than those received first/second-generation ones (99.3% vs. 94.7%, *p* = 0.034). Following multivariate analysis, only the type of DAA regimen used emerged as being the only independent variables statistically associated with treatment failure (OR 7.889, 95%CI 1.288–48.335; *p* = 0.026). Despite this, all patients who relapsed were subsequently retreated with sofosbuvir/velpatasvir/voxilaprevir and obtained SVR.

### 3.3. Long-Term Follow-Up

All participants who achieved SVR represented the population at risk of reinfection. The median follow-up time after EOT was 53 weeks (range 10–260). During this time, we identified seven reinfections, yielding a reinfection rate of 1.25/100 PY. The median estimated time from EOT to reinfection was 24 weeks (range 12–64), with six (85.7%) cases detected during the first year of follow-up. Reinfection was associated with age at treatment ≤50 years (85.7%), male sex (85.7%), receiving OST (100%), recent drug use (71.4%), and history of alcohol abuse (85.7%). Following multivariate analysis, recent drug use before treatment was found to be the only independent predictor of HCV recurrence (OR 11.07, 95%CI 2.10–58.38; *p* = 0.005). In fact, the incidence of reinfection was 5.3/100 PY among recent PWID and 0.4/100 PY among former ones (*p* < 0.001).

Despite virologic response, among 166 patients with advanced fibrosis, an occurrent HCC was diagnosed in four, patients and a recurrent HCC in another two. In our analysis, the HCC incidence rate was 1.5/100 PY. No additional patients presented with decompensated cirrhosis. Death occurred in two patients, one caused by extrahepatic cancer and one due to suicide via a drug overdose.

## 4. Discussion

The introduction of DAA-based therapy for CHC has profoundly changed the landscape of this liver disease, allowing the possibility to eradicate the infection in almost every patient. Access to HCV treatment represents a crucial step in reducing the burden of this infection. However, it is still poor for PWID who represent the majority of incident (80%) and prevalent (60%) cases [14]. HCV-infected PWID are often unaware of their infection, usually have difficulties in reaching public health services, and only one in ten of those diagnosed enter treatment [15]. Frequently, these kinds of patients are reluctant to accept medical help, and despite being aware that they are HCV-antibody positive, they do not undergo further evaluation or do not keep appointments [16,17]. Furthermore, they do not like to be referred to the hospital but prefer being treated in SerDs.

The model we propose in this study could be a highly relevant strategy to engage patients in care, as it allows a rapid characterization of liver disease and a rapid access to antiviral treatment, reducing the risk of patients being lost. In our experience, PWID were quickly started on DAA following a clinical evaluation. Our approach with systematic ultrasound evaluation in a setting of subjects with multiple liver injury factors (such alcohol abuse and high BMI) has allowed us to identify patients with advanced fibrosis/cirrhosis at risk of HCC development. In fact, an advanced fibrosis was assessed in about 50% of subjects and HCC was diagnosed early and treated with local ablation in two patients. However, despite these findings, the vast majority of patients were treatment naive (about 80%). The main reason was the high prevalence of psychiatric disorders (42.8%): in the past, previous standard treatments with interferon were contraindicated. Moreover, many patients refused the interferon-based therapy, as they feared the occurrence of side effects or were experiencing social problems, such as unemployment, active alcoholism, or living alone.

In agreement with a recent systematic review on the global distribution of genotypes in PWID [18], we confirmed that genotypes 1a and 3 are more prevalent among substance users, driving emerging trends in the HCV epidemic.

Almost all HCV-infected PWID referred to our SerDs were promptly treated, showing a good compliance to therapy. The high rate of SVR reported in both clinical trials and real-life observations around the world [6,7,19,20,21,22,23,24] was also confirmed in our study. Moreover, our results corroborate those reported in a similar experience that prospectively recruited about 1500 PWIDs inside SerDs distributed all over Italy [25]. The authors showed a significantly higher SVR rate and treatment adherence among PWID followed in the SerDs group than those followed directly by liver centers. These data are mainly due to the success of this organizational system and underline the pivotal role of the close cooperation between the SerDs and territorial hepatologists centers in the high SVR achievement.

In our cohort, most of the virologic failure was driven not by patients who relapsed, but by those who prematurely discontinued or were lost to follow-up. Overall, in the mITT population, SVR rate was very high (98.5%). Furthermore, patients lost during follow-up without available SVR data completed the planned treatment duration and would probably have achieved SVR. Virologic failure in OST patients did not significantly differ from non-OST (6.5% vs. 3.6%; *p* = 0.273), confirming the growing body of evidence from others real-world studies [6,19,20,21,22,23].

On the contrary, when recent PWID were compared to former ones, there was a clinically significant difference in SVR attainment, even if drug use could be an underestimation, since these data were self-reported and not assessed by scheduled urine testing. The association between recent drug use and treatment failure was also confirmed by multivariable logistic regression models. It is likely that PWID identified as recent drug users could be those with more disruptive behavior associated with drugs, leading to drop-out. This is in contrast with a recently published meta-analysis [26], including more than 3500 patients that demonstrated that people reporting recent drug use achieved similar SVR rates compared to former drug users.

The high SVR rate could also be explained by the fact that most patients were treated with pangenotypic third-generation drugs. In fact, the only independent predictor of virological failure found in the mITT population was the treatment received, most likely due to the higher antiviral potency and genetic barrier of the third-generation compared to first- and second-generation regimens. During the study, glecaprevir/pibrentasvir association for 8 weeks was approved by the Italian Drug Regulatory Agency for patients without cirrhosis, regardless of HCV genotype. Today, the availability of a shorter duration schedule weeks also in naive patients with compensated cirrhosis [27] could further facilitate the adherence to medical visits and the successful treatment completion. Additional studies would be needed to confirm the benefit of shorter treatment durations on efficacy and adherence in this setting. In agreement with the safety profile documented in other real-life studies [6,19,20,21,22,23,25,28], all treatments were well tolerated with low incidence of adverse events and no SAEs and compliance was excellent. This was also possible thanks to the availability of ribavirin (RBV)-free schedules, thus preventing adverse events, such as anemia and asthenia. In fact, in our study, 56 (15.6%) patients were addicted to RBV, mainly associated with first/second-generation regimens.

Finally, we reported an overall reinfection rate after successful HCV treatment of 1.25/100 PY. Many authors have reported higher reinfection rates among PWID compared with our cohort [26,29,30,31]. This may be attributed to the high heterogeneity of populations across studies, including differences in risk profile, ongoing injecting drugs use, and multiple co-occurring conditions, such as alcohol abuse and HIV co-infection. Our data are closely in line with those reported in a large population-based registry of more than 2000 DAA-treated patients [32]. Similar to this study, most reinfections occurred within one year of SVR. The low HCV reinfection rate in our cohort may reflect the relatively short median follow-up time, but also the positive impact of our integrated model of care on patient’s behavior changes following HCV treatment.

However, the reinfection rate increases up to 5.3/100 PY when we analyzed only recent PWID. This finding is more in keeping with those reported in a recent meta-analysis where the pooled rate among people reporting recent injecting drug use was 6.2/100 PY [33].

This study has several limitations. The PWID enrolled likely represent a selected population engaged in their healthcare, and therefore these findings may not be generalizable to other populations of PWID not managed in the SerDs. Another limitation that reduces the generalizability of this analysis to the wider population is the exclusion of patients co-infected with HIV, which has a high prevalence among PWID. However, in clinical practice, HIV individuals shows a similar SVR rate than in individuals not infected with HIV. Finally, the number of recent PWID included in this study is small and makes it difficult to draw definitive conclusions on the association of this factor with a higher rate of virologic failure and reinfection.

## 5. Conclusions

In conclusion, HCV treatment delivered to PWID within our multidisciplinary program of care leads to very high SVR rates and treatment adherence and a low rate of drop-out. The results of this study indicate that DAA treatment, especially using the third-generation regimen, is highly effective and safe in PWID, supporting current guideline recommendations to prioritize access to HCV treatment in this vulnerable population, which includes a high number of transmitters of the infection. Approaches such as ours, in which hepatologists directly collaborate with SerDs healthcare workers for treatment and follow-up in the context of an integrated model, could maximize HCV cure for PWID and contribute to long-term maintenance in care. However, long-term follow-up studies are needed to investigate the probability of reinfection and the impact on overall morbidity and mortality in PWID, in particular in recent drug users. Moreover, an advantage of this approach is also the correct staging of liver disease with elastography and the ultrasound monitoring of patients also with other causes of liver disease. In this care setting, a global evaluation of patients is mandatory, and not only for HCV treatment. If this integrated model for PWID is also found to be cost-effective compared to the standard care, it could be worthwhile to extend this model nationwide.

## Figures and Tables

**Table 1:** Characteristics of PWID with CHC started treatment.

Characteristics	All Patients (*n* = 358)
Age (years)	49 (18–69)
Birth cohort:	
● <1965	87 (24.3%)
● 1965–1974	171 (47.8%)
● ≥1975	100 (27.9%)
Male sex	297 (83%)
BMI ≥ 25	179 (50%)
Advanced fibrosis	176 (49.1%)
Fibrosis stage:	
● F0–1	124 (34.6%)
● F2	58 (16.2%)
● F3	66 (18.4%)
● F4	110 (30.7%)
Genotype:	
● 1a	149 (41.1%)
● 1b	21 (5.9%)
● 2	2 (0.6%)
● 3	142 (39.7%)
● 4	44 (12.3%)
HCV treatment naive	281 (78.5%)
HCV treatment schedule:	
● First generation	7 (2%)
● Second generation	52 (14.5%)
● Third generation	299 (83.5%)
HCV treatment regimen:	
● Glecaprevir/pibrentasvir	234 (65.4%)
● Sofosbuvir/velpatasvir	54 (15.1%)
● Elbasvir/grazoprevir	11 (3.1%)
● Ombitasvir/paritaprevir/ritonavir/dasabuvir	15 (4.2%)
● Ombitasvir/paritaprevir/ritonavir	8 (2.2%)
● Sofosbuvir/daclatasvir	20 (5.6%)
● Sofosbuvir/ledipasvir	9 (2.5%)
● Sofosbuvir/simeprevir	3 (0.8%)
● Sofosbuvir	4 (1.1%)
HBV co-infection	4 (1.1%)
Diabetes	20 (5.6%)
Arterial hypertension	41 (11.5%)
Psychiatric disorders	140 (39.1%)
Psychiatric treatment:	
● Benzodiazepine	104 (29.1%)
● Antipsychotics	72 (20.1%)
● Antidepressants	34 (9.5%)
● Valproic acid	12 (3.4%)
Any alcohol use	259 (72.3%)
Current tobacco use	296 (82.7%)
OST prescription:	
● Methadone	204 (57%)
● Buprenorphine	43 (12%)
Drug-use status:	
● Former	284 (79.3%)
● Recent	74 (20.7%)

CHC, chronic hepatitis C; BMI, body mass index; HCV, hepatitis C virus; HBV, hepatitis B virus; OST, opioid substitution treatment.

**Table 2:** Intention-to-treat SVR rate according to genotypes and antiviral treatment regimens.

Antiviral Treatment Regimens	SVR Rate					
	Genotype 1a	Genotype 1b	Genotype 2	Genotype 3	Genotype 4	Total
**First-generation regimens**	2/2 (100%)		1/1 (100%)	2/3 (66.7%)	0/1 (0%)	5/7 (71.4%)
Sofosbuvir + RBV			1/1 (100%)	2/3 (66.7%)		3/4 (5%)
Sofosbuvir/Simeprevir ± RBV	2/2 (100%)				0/1 (0%)	2/3 (66.7%)
**Second-generation regimens**	18/18 (100%)	3/4 (75%)		18/20 (90%)	10/10 (100%)	49/52 (94.2%)
Ombitasvir/Paritaprevir/Ritonavir/Dasabuvir ± RBV	11/11 (100%)	3/4 (75%)				14/15 (93.3%)
Ombitasvir/Paritaprevir/Ritonavir ± RBV					8/8 (100%)	8/8 (100%)
Sofosbuvir/Daclatasvir ± RBV				18/20 (90%)		18/20 (90%)
Sofosbuvir/Ledipasvir ± RBV	7/7 (100%)				2/2 (100%)	9/9 (100%)
**Third-generation regimens**	123/128 (96.1%)	16/17 (94.1%)	1/1 (100%)	112/119 (94.1%)	32/33 (97%)	284/299 (95%)
Glecaprevir/Pibrentasvir	106/111 (95.5%)	9/10 (90%)	1/1 (100%)	82/87 (94.3%)	24/25 (96%)	222/234 (94.9%)
Sofosbuvir/Velpatasvir ± RBV	14/15 (93.1%)	1/1 (100%)		30/32 (93.8%)	6/6 (100%)	51/54 (94.4%)
Elbasvir/Grazoprevir ± RBV	3/3 (100%)	6/6 (100%)			2/2 (100%)	11/11 (100%)
**Total**	143/149 (96%)	19/21 (90.5%)	2/2 (100%)	132/142 (93%)	42/44 (95.5%)	338/358 (94.4%)

SVR: sustained virologic response; RBV: ribavirin.

**Table 3:** Factors associated with failure treatment (according ITT analysis) to direct acting antiviral drug combinations.

				Multivariate Analysis	
Variable	n/N	SVR	*p*	Odds Ratio (95%IC)	*p*
Age:					
● <50 years ● ≥50 years	203/216 135/142	94% 95.1%	0.661		
Sex:					
● Male ● Female	280/297 58/61	94.3% 95.1%	0.803		
Stage of fibrosis:					
● Mild/moderate ● Advanced/cirrhosis	172/182 166/176	94.5% 94.3%	0.939		
HCV genotype:					
● Genotype 3 ● Others genotypes	132/142 206/216	93% 95.4%	0.331		
Previous HCV treatment:					
● Naive ● Experienced	265/281 73/77	94.3% 94.8%	0.866		
Alcohol use:					
● Yes ● No	243/259 95/99	93.8% 96%	0.431		
OST					
● Yes ● No	231/247 107/111	93.5% 96.4%	0.273		
Recent drug use:					
● Yes ● No	66/74 272/284	89.2% 95.8%	0.028	2.747 (1.080–6.992)	0.034
Psychiatric disorders:					
● Yes ● No	136/140 202/218	97.1% 92.7%	0.072		
HCV treatment received:					
● First/second generation ● Third generation	54/59 284/299	91.5% 95%	0.291		

SVR, sustained virologic response; ITT, intention-to-treat; HCV, hepatitis C virus; OST, opioid substitution treatment.

## Data Availability

Not applicable.

## Supplementary Materials

The following are available online at https://www.mdpi.com/article/10.3390/jcm10174001/s1, Table S1: Characteristics of PWID with CHC started treatment according drugs use.
