# Supplementary material for: Model of Care for Microelimination of Hepatitis C Virus Infection among People Who Inject Drugs
Source: J Clin Med. 2021 Sep 3;10(17):4001. doi: 10.3390/jcm10174001 (PMC8432451; doi:10.3390/jcm10174001)
Supplement: Supplementary file 1 [file jcm-10-04001-s001.zip › jcm-1346212-supplementary.pdf]

Table S1. Characteristics of PWID with CHC started treatment according drugs use.

Characteristics	All patients (*n* = 358)	Former PWID (*n* = 284)	Recent PWID (*n* = 74)	p
**Age > 50**	142 (39.7%)	125 (44%)	17 (23%)	0.001
**Male sex**	297 (83%)	231 (81.3%)	66 (89.2%)	0.110
**BMI ≥ 25**	179 (50%)	152 (53.5%)	27 (36.5%)	0.009
**Advanced fibrosis**	176 (49.1%)	146 (51.4%)	30 (40.5%)	0.096
**Genotype:**				
• 1a	149 (41.1%)	110 (38.7%)	39 (52.7%)	
• 1b	21 (5.9%)	17 (6%)	4 (5.4%)	
• 2	2 (0.6%)	1 (0.4%)	1 (1.4%)	
• 3	142 (39.7%)	123 (43.3%)	19 (25.7%)	0.063
• 4	44 (12.3%)	33 (11.6%)	11 (14.9%)	
**HCV treatment naive**	281 (78.5%)	220 (77.5%)	61 (82.4%)	0.354
**Treated with third generation regimen**	299 (83.5%)	231 (81.3%)	68 (91.8%)	0.029
**HBV coinfection**	4 (1.1%)	4 (1.4%)	0	0.585
**Diabetes**	20 (5.6%)	19 (6.7%)	1 (1.4%)	0.090
**Arterial hypertension**	41 (11.5%)	35 (12.3%)	6 (8.1%)	0.413
**Psychiatric disorders**	140 (39.1%)	108 (38%)	32 (43.2%)	0.425
**Any alcohol use**	259 (72.3%)	201 (70.8%)	58 (78.4%)	0.193
**Current tobacco use**	296 (82.7%)	232 (81.7%)	64 (86.5%)	0.331
**OST prescription**	69%247 ()	180 (63.4%)	67 (90.5%)	<0.001

CHC: chronic hepatitis C; BMI: body mass index; HCV: hepatitis C virus; HBV: hepatitis B virus; OST: opioid substitution treatment.
